# Travelling Editorials

**Published:** 1843-08

**Authors:** 


### THE WESTERN JOURNAL.

Vol. VIII.—No. II.

LOUISVILLE, AUGUST 1, 1843.

### TRAVELLING EDITORIALS.

### *Voyage up the Mississippi.*

He who would enjoy the ascending voyage on this river, should embark at New Orleans in the month of June, when the river is in its glory, and its banks are concealed beneath leaves more numerous than the sands of its waters. At this stage, the steamer moves fearlessly over her great enemy, the sawyer, and has as little dread of shoals and sand bars; all of which lie quiet and harmless, in the depths of the mighty flood. But greater safety is not the only advantage. There is a physical pleasure in coming every hour to a higher level and a higher latitude, which conspire to give a cooler and fresher climate. Still further, the perception of rapid progress against such an impetuous torrent, raises ones race in his estimation, and sets him to speculating on the future conquests of man over the elements, before which, in a state of nature, he is so powerless. Further still, the geologist sees the turbid waters overflowing the banks to deposit, on the adjoining plains, the alluvion which has not yet been raised high enough to confine the stream within its borders; while the physician beholds in the same phenomenon the conditions originating the fevers which, after the subsidence of the freshet, invade the inhabitants of the great valley Finally, at such a season the Father of Waters is not in *dishabille*, but his sunken trees, and crumbling banks, and desolate bars overspread with the decaying wreck of the forest, are nearly all concealed, and little arrests the eye, but trees and water—a mighty river sweeping through a lofty and boundless wood; of which the rivulet meandering in the meadow is a microscopic picture. On the whole, these views are monotonous but not tiresome, similar but not identical. In some places the gigantic cotton trees range up to the water's edge; in others, the eye reaches them over three or four green terraces, rising in soft beauty from the surface of the stream. Below the latitude of 33° their branches are gloomily decorated with dark silvery Spanish moss; above, the trunks are encircled with rhus and ampylopsis, like the columns of a mighty temple, festooned with wreaths of evergreen. But in the midst of uniformity the bayous give great and pleasing variety. Every now and then the voyager is surprised to find himself, as if by magic, suddenly translated from the broad surface of the eddying river, to a narrow and winding canal, into which the lower limbs of the trees dip their leaves; while the upper cast a refreshing shade over the hurricane deck, on which, if a lover of nature, he will spend much of his time, and where, as the pleasantest spot we can select for him this sultry afternoon, we shall leave him to lounge, and look at what we have not described.

*The Influenza.*

From what we can see and hear, this new visitation of epidemic catarrh is extensively felt over the "Ohio countries." As in the case of epidemic cholera, appearing first in the larger towns, it subsequently invades even the most thinly peopled portions of the country. The newspapers inform us of its simultaneous existence in Europe and on the Atlantic ocean. In the United States it has manifestly spread from east to west, as did the cholera before it. Indeed the laws of diffusion of the remote causes of these two epidemics, appear to be the same; and they agree pathologically in one point, that of attacking chiefly the mucous membranes, one the respiratory, the other the digestive. The effects of the reigning influenza are not however limited to the pulmonary and facial mucous membrane; for in a great number of cases the gastro-intestinal has been and is, the seat of irritation, giving rise to cholera morbus, cholera infantum, diarrhœa, and dysentery. It must, however, be borne in mind, that these maladies prevail annually in July, and would no doubt occur to a greater or less extent if the influenza did not prevail at this time. Nevertheless, the causes of the whole are in concurrent operation, so that their symptoms in many cases are mixed up, or appear in succession. The present influenza, considered apart from its accomplices, is not as violent as some which have preceded it; and occurring at midsummer, is not so likely to leave serious lesions of the lungs behind it, as when it occurs in autumn, winter, and spring.

We shall fill out this jejune editorial with an extract from the private letter of a respected medical gentleman, dated Detroit, Michigan, April 15, 1843.

"I am looking for an epidemic summer. Notwithstanding M. Arago's opinions with regard to the influence of comets, I am still persuaded of the truth of Webster's remarks. My own experience, though necessarily so limited, fully bears him out. If a comet is to influence us, I take it that as a necessary condition, it must either be so large or so near as to be distinctly visible to the naked eye. Such comets, I believe, have always been accompanied with earthquakes, irruptions of volcanoes, and severe seasons of epidemics; I mean to those nearest the meteor's perihelion. The first we have had, the last I certainly look for. Analogy would teach, that their influence (epidemic) would diminish with the square of their distance, or the cube of their matter. Arago's two comets a year, are too small and too distant to have any effect. We might as well expect cometary influence from those bodies dispersed through space, as to look for such when the meteor is invisible."

The occurrence in summer, after an earthquake in winter and a comet in spring, of a wide spreading epidemic, dependent on no known or even conjectural terrestial or atmospheric cause, certainly gives support to the speculation of our correspondent. He may almost be said to have predicted the present epidemic. We hope our readers in the different parts of the Mississsppi valley, will note accurately the time of its invasion, its symptoms and complications, that we may have its chronology and pathology, should its ætiological history still remain unwritten.

*Reported Yellow Fever in Pittsburg.*

An esteemed correspondent of the neighborhood of Pittsburg, informs us that a report has reached him of two cases of yellow fever in that city. If they have occurred, the patients had no doubt contracted the disease in New Orleans. We refer to the rumor only to say, that the question of the contagiousness of yellow fever will probably be answered in the valley of the Mississippi. The rapidity with which steamboats ascend into the interior from that city, cannot fail to introduce the disease into many of our commercial towns, *if it be contagious.*

*Progress of Mesmerism.*

We have cut the following important annunciation from the Cincinnati Daily Gazette of the 27th ult:

"*Animal Magnetism.*—Dr. S. Underhill, of Cleveland, Ohio, late Professor of Chemistry, who was appointed in 1839 by the State Medical Society, one of a committee of three, to investigate the above subject, and who has devoted much of his time, since that appointment, to the subject, has arrived in this city and intends to deliver lectures and make demonstrations on this and on Phrenology. He claims much experience in the use of Magnetism as a curative agent. He has with him, at the "Mansion House," a somnambulist, whose ability to find and point out the seat, character, and sympathetic effects of any disease however mysterious, have been fully tested and have never failed in a single instance. During the last three months physicians have daily taken notes of her examinations, and in no case has she made a failure. Her prescriptions are most appropriate for the disease described. Price for examination and prescription two dollars. Always private; none present except the physician, wife, husband or parents of the patient.

"Of the when and where lectures will be delivered, due notice will be given."

It appears from this advertisement that Dr. Underhill is a physician of the largest city in the north of Ohio, a professor of chemistry, and a member of a committee of three, selected and appointed by our brethren in Ohio to investigate the subject of animal magnetism. He is then not an unbidden, or "come by chance" member of the Mesmeric family, but a legitimate and commissioned operator, whom we may venture to "touch without tongs," though perhaps not without being soiled either by the contact or by having "mud thrown upon us," by the good gentlemen (bless their souls)! who are desirous of paying "two dollars" to a pretty young girl for looking into them.

What a pity it is that the Doctor cannot bestow *clairvoyance* on himself, for he would then pocket the whole fee (though perhaps he prefers half for the sake of such an interesting travelling companion), and would, moreover, have the pleasure of seeing many exciting sights, while a certain class of patients would doubtless derive a pleasure from his inspection, which they cannot from that of one of their own sex. The perfection of the arrangement would be for the Doctor and his fair partner in the practice both to inspect, dividing the applicants between them; thus gratifying their own philosophical curiosity, while they afforded to their patients the satisfaction of being inspected by one of the other sex from themselves. Thus methodized, the Mesmeric practice of physic could not fail to become exceedingly popular. Moreover, by dividing the sexes between them, each would soon become more *au fait* than could otherwise happen. The Doctor could decide, at a glance, on the state of the *ovaria*, count the number of *corpora lutea*, detect obstructions in the *tubuli Fallopianæ*, spy out the *embryo* in its first "swaddling clothes," and distinguish *leucorrhœa* from every other discharge; while his fair assistant would as infallably diagnosticate *gonorrhœa* from *gleet*, organic from spasmodic *strictures*, and *sarcocele* from every other tumour, without any indecent exposure in her presence, or any befouling of her delicate hands. Thus practised, medicine would become a delicious occupation, bringing its own reward, even without the "two dollars"—while half the affliction from the disease would be swallowed up, in the pleasure of being looked into by one of the other sex.

But what is to become of our medical schools under this mighty discovery? We fear that our professors, like the handicraft artists of Europe, when labor-saving machinery was first introduced, will rise *en masse*, and break it down. What a pity that the public spirited citizens of Louisville expended a hundred thousand dollars to endow our medical institute, when such a brilliant invention for the practice of physic was almost peeping above the horizon. Had they waited only a few years, they might have saved their money for some *necessary* work of benevolence. We would advise our *alumni* and all other young doctors to toil no more over their books, but go to Mesmerizing the young ladies of their neighborhood, and as soon as they find one who is "impressible," to marry and commence the new practice. It will be every way pleasanter to ride about the country with a chatty little wife, whose *clairvoyance*, physical and *intellectual*, will penetrate all obscurities in diagnosis, pathology and therapeutics, than to trudge alone to the house of a patient, and then prescribe perhaps in hesitancy and error.

In conclusion, we protest against being claimed as an implicit and unconditional believer in Mesmerism, because we have commended *one* of its applications. We believe largely, it is true, and our mental *clairvoyance* is on the increase, but it may be sometime before we perceive as good evidence for some of the other Mesmeric achievements as for that which we have made known to our readers.

P. S. Having spoken of self Mesmerization, we may cite a fact to show its reality. In Cincinnati a lad was so impressible, that he could readily throw himself into a somnambulic state. This he did in presence of a committee of highly respectable gentlemen, two of whom assured us of the fact. Having remained for some time in that condition, the question arose, how he should be brought out of it. After considerable deliberation it was resolved that two of their body should take hold of his arms and make the reverse or disenchanting passes. As his arms were stiff, this proved to be rather an awkward movement, and in the midst of it, he pushed them away exclaiming: "get along, you don't know nothing about it, I can do much better myself;" and waving his hands *secundum artem* a few times, was wide awake! This discovery in Mesmerism seems especially adapted to the practice of physic, and will no doubt soon be applied. The physician, on the present plan, when he takes his seat at the bed side of the patient, is obliged to keep awake, a pretty difficult matter if the thermometer be as it now is above 90°, but on the improved plan, he has only to sit down and throw himself into a Mesmeric slumber, when he will infallibly see what is and what should be; after which he may by the reverse passes wake himself up, and proceed to execute. But one step more is necessary. To proceed from self to reciprocal Mesmerization. Thus, as in the case of Dr. U., where a man and woman operate in connexion, if they were able mutually to impregnate each other with the influence which begets knowledge and science, not less than corporeal *clairvoyance*, they might explore in conjunction what we once heard an old professional brother call the "secret arcanas" of the patients structure, and by comparing their observations come to a more infallible conclusion than if acting separately. The obvious pleasures and advantages of this method, lead us respectfully to suggest to our Mesmeric societies the propriety of offering a premium to the man who might discover a process of reciprocal Mesmerization; and to aid in the momentous inquiry, we would direct the attention of competitors for the prize to certain worms, or other small animals, which are said to carry on reciprocal impregnation. But lest our *postcript* should exceed the length of the text to which it is appended, we must, for the present, dismiss this fascinating subject. D.

*Scioto Valley, Ohio,* July 25, 1843.

---

